# Characteristic immunophenotype and gene co-mutational status orchestrate to optimize the prognosis of *CEBPA* mutant acute myeloid leukemia

**DOI:** 10.1038/s41408-023-00838-2

**Published:** 2023-05-05

**Authors:** Xinjie Chen, Diyaer Abuduaini, Yuliang Zhang, Jun Long, Xiaojing Lin, Hongming Zhu, Jianfeng Li, Yang Shen

**Affiliations:** 1 Shanghai Institute of Hematology, State Key Laboratory of Medical Genomics, National Research Center for Translational Medicine at Shanghai, Ruijin Hospital, Shanghai Jiao Tong University School of Medicine, Shanghai, China; 2 Department of Hematology, The Second People’s Hospital of Kashi, Kashi, Xinjiang China; 3 Department of Hematology, Tongji Hospital, Tongji University School of Medicine, Shanghai, China

**Keywords:** Acute myeloid leukaemia, Disease-free survival

**Dear Editor**,

Mutation on *CEBPA* (*CEBPA*^*mut*^) is one of the most common molecular abnormalities in acute myeloid leukemia (AML), especially in east Asian population [1]. As recently reported, in-frame mutations in bZIP domain of CEBPA (*CEBPA*^*bZIP-inf*^) exerted higher potency in favorable-risk prediction than biallelic mutated *CEBPA* (*CEBPA*^*bi*^), although cases were highly overlapped between the two categories [2–4]. However, about 30–50% *CEBPA*^*bi*^ AML cases consolidated with chemotherapy alone suffered from disease relapse [5, 6], retaining the same *CEBPA*^*mut*^ patterns as diagnosis [7, 8]. Hence, there might be clinically and biologically heterogeneous under current context of *CEBPA*^*mut*^ grouping, and a comprehensive assessment of *CEBPA*^*mut*^ AML prognosis remains to be established.

In this study, a total of 293 *de novo CEBPA*^*mut*^ AML patients were enrolled, with biological data available in 124 patients (Supplementary Fig. 1A). Usually, *CEBPA*^*bZIP-inf*^ AML patients were diagnosed at younger age, with higher white blood cell counts, hemoglobin levels and lower platelet counts compared with other *CEBPA*^*mut*^ AML patients (*CEBPA*^*other*^); while risk classification of karyotypes according to the ELN 2022 showed no differences between *CEBPA*^*bZIP-inf*^ and *CEBPA*^*other*^ AML (Supplementary Table 1).

Consistence with previous reports [2, 3], *CEBPA*^*bZIP-inf*^ AML correlated with higher CR rate (Supplementary Table 1), which could translate into improved overall survival (2-year OS: 86% vs. 53.1%, *p* = 0.0019) and event-free survival (2-year EFS: 64.7% vs. 37.5%, *p* = 0.01) (Supplementary Fig. 1B). However, the OS, EFS and relapse rate (Supplementary Table 1) of the patients who achieved CR showed no difference between *CEBPA*^*bZIP-inf*^ and *CEBPA*^*other*^ AML (2-year OS: 87% vs. 63.1%, *p* = 0.07; 2-year EFS: 65.5% vs. 47.7%, *p* = 0.19) (Supplementary Fig. 1C). It seemed that the current risk stratification based on *CEBPA*^*mut*^ locus could not sufficiently distinguish certain patients who may develop disease progression.

Most patients with *CEBPA*^*bZIP-inf*^ (85/89, 95.5%) displayed cross-lineage expression of CD7, while only 20/35 (57.1%) patients with *CEBPA*^*other*^ harboring CD7-positive immunophenotype (*p* < 0.001) (Supplementary Table 1). CD7-positive cases showed distinct gene expression patterns compared with CD7 negative cases (Supplementary Fig. 2). Survival analysis further indicated CD7 could significantly distinguish the clinical outcome in the whole cohort of 293 *CEBPA*^*mut*^ AML cases, with improved 2-year OS and EFS of 81.8% and 66.4% respectively in CD7-positive *CEBPA*^*mut*^ AML vs. 48.8% and 33.0% respectively in CD7-negative *CEBPA*^*mut*^ AML (*p* < 0.0001 and <0.0001; Supplementary Fig. 3A, B). Moreover, CD7-negative *CEBPA*^*mut*^ AML patients also had a shorter disease-free survival compared with CD7-positive patients (2-year DFS: CD7-positive *CEBPA*^*mut*^ AML 63.1% vs. CD7-negative *CEBPA*^*mut*^ AML 39.4%, *p* < 0.0001; Supplementary Fig. 3C).

Given the prognostic significance of CD7 in *CEBPA*^*mut*^ AML, survival analysis of 117 CR patients demonstrated that the combine of CD7 with the *CEBPA*^*mut*^ locus could discriminate disease prognosis, with distinguished 2-year OS and EFS: *CEBPA*^*bZIP-inf*^/CD7 + AML, 90.4% and 68.8% vs. other *CEBPA*^*mut*^ AML, 56.6% and 41.3%, respectively (*p* < 0.0001 and = 0.0076; Supplementary Fig. 3D, E). Besides, the 2-year DFS of *CEBPA*^*mut*^ AML was as follows: *CEBPA*^*bZIP-inf*^/CD7 + AML 72.5% vs. other *CEBPA*^*mut*^ AML 40.6%, (*p* = 0.0028; Supplementary Fig. 3F). Multivariable analysis further confirmed *CEBPA*^*bZIP-inf*^/CD7+ as an independent risk factor that favors the prognosis of *CEBPA*^*mut*^ AML, with hazard ratio of 0.16 (*p* = 0.001), 0.45 (*p* = 0.034), and 0.39 (*p* = 0.018) in OS, EFS and DFS, respectively (Supplementary Table 2).

The distribution of co-mutations was illustrated in Fig. 1A. The co-mutations of the 117/124 CR patients were categorized into Transcriptional Factors (TFs, 31/117, 26.5%), Chromatin/Cohesion/Spliceosome (CCS, 54/117, 46.2%), Receptor Tyrosine Kinases (RTKs, 49/117, 41.9%), Tumor Suppressor (TS, only *WT1* mutation in this group, 23/117, 19.7%) and Nucleolar (only *NPM1* mutation in this group, 7/117, 6.0%). Notably, *CEBPA*^*bZIP-inf*^/CD7 + AML were more frequently accompanied with mutations in TFs than other *CEBPA*^*mut*^ AML (32.1% vs. 12.1%, *p* = 0.027). Whereas mutations in CCS were highly enriched in other *CEBPA*^*mut*^ AML compared to *CEBPA*^*bZIP-inf*^/CD7 + AML (60.6% vs. 40.5%, *p* = 0.049). No Nucleolar (*NPM1*) mutations were found in *CEBPA*^*bZIP-inf*^/CD7 + AML, while 21.2% of the rest *CEBPA*^*mut*^ patients harboring *NPM1* mutations (*p* < 0.001; Supplementary Table 3).

**Fig. 1:**
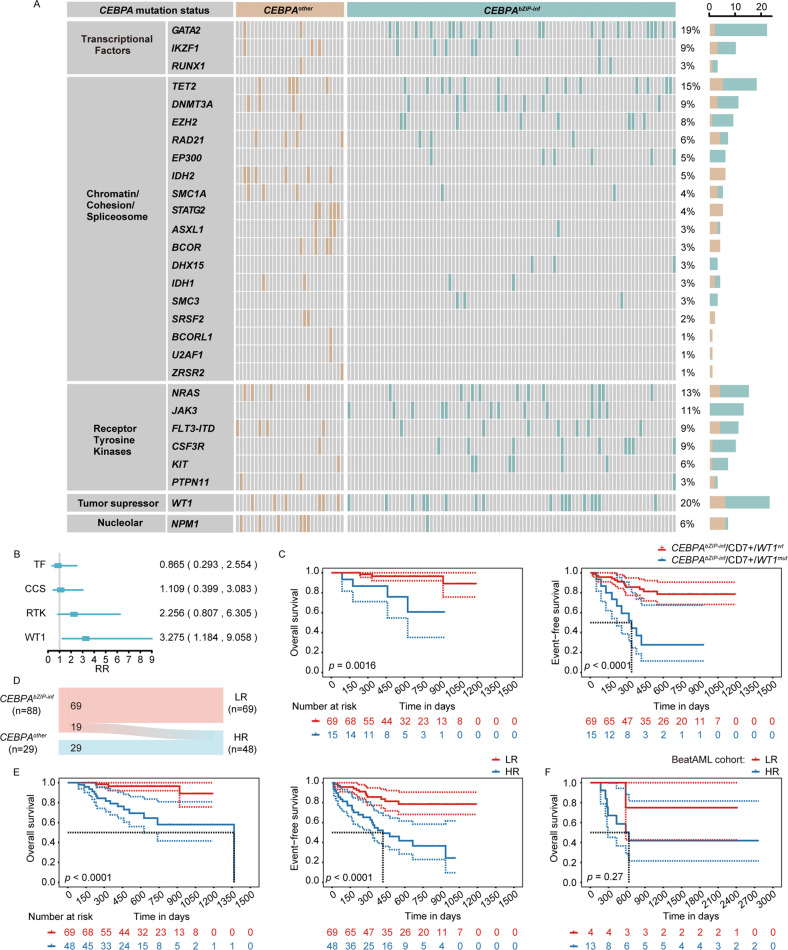
Integrating CD7 expression and WT1 mutation status for revised risk stratification of *CEBPA*^*bZIP-inf*^ AML patients. **A** The distribution of co-mutations within the cohort of 124 *CEBPA*^*mut*^ AML patients. Genes were categorized into groups as labeled on the left. **B** Cox-proportional hazard regression analysis for the categorized co-mutations independently affecting OS of 117 CR-achieved *CEBPA*^*mut*^ AML patients. TF transcriptional factor, CCS chromatin/cohesion/spliceosome, RTK receptor tyrosine kinase, TS tumor suppressor. **C** Kaplan–Meier curves for the survival of 84 CR-achieved *CEBPA*^*bZIP-inf*^/CD7+AML patients according to *WT1* mutation status. **D** Sankey plot for reclassification of 117 CR-achieved *CEBPA*^*mut*^ AML patients from *CEBPA*^*bZIP-inf*^/*CEBPA*^*other*^ grouping to the revised risk stratification. **E** Kaplan–Meier curves for the survival of 117 CR-achieved *CEBPA*^*mut*^ AML patients according to the revised risk stratification. **F** Kaplan–Meier survival curves for OS of 17 *CEBPA*^*mut*^ AML patients within the BeatAML cohort according to the revised risk stratification.

The corresponding clinical impacts of co-mutations were involved into prognosis evaluation. We categorized the co-mutations into groups (CCS, RTKs, TS, Nucleolar) to avoid the interference of low-frequency mutations as independent variables for hazard analysis. Multivariate Cox regression analysis showed TS (*WT1* mutations, *WT1*^*mut*^) significantly affected the OS of *CEBPA*^*mut*^ AML, with risk ratio (RR) of 3.275, *p* = 0.0223 (Fig. 1B). Further analysis indicated *WT1*^*mut*^ could significantly shorten the survival of *CEBPA*^*bZIP-inf*^/CD7 + AML, with 2-year OS and EFS of 96.6% and 78.6% vs. 60.7% and 27.8%, respectively (*p* = 0.0016 and <0.0001, respectively) (Fig. 1C).

Net reclassification improvement (NRI) was then performed and indicated that the outcome was significantly improved after the integration of CD7 expression and *WT1* status into the clinical nomogram, with the value of 39.2% improvement [95% CI: 0.000–1.059]. Thus, we defined *CEBPA*^*bZIP-inf*^ AML patients characterized by immunophenotypic CD7-positive and wild-type *WT1* as low-risk group (LR group: *CEBPA*^*bZIP-inf*^/CD7 + /*WT1*^*wt*^); correspondingly, 19/89 patients of *CEBPA*^*bZIP-inf*^ AML were re-stratified into the high-risk group (HR group, Fig. 1D). With the revised stratification, patients in LR group had a superior outcome than the patients in HR group (2-year OS: 96.6% vs. 64.4%, *p* < 0.0001; 2-year EFS: 78.6% vs. 36.6%, *p* < 0.0001; Fig. 1E). We also validate the revised stratification in patients from BeatAML cohort. Improved OS was observed in LR patients (*n* = 4) compared with HR patients (*n* = 13), although the difference was not significant due to the limited sample size (*n* = 17, Fig. 1F).

The transcriptomic data was available in 122 (data of 2 patients were missing) *CEBPA*^*mut*^ patients from our cohort. With unsupervised cluster analysis, *HOXA/B* family genes were identified to be highly associated with poor prognosis in *CEBPA*^*mut*^ AML (Fig. 2A). Besides, the differentially expressed genes (DEGs) analysis also showed that *HOXA/B* family genes were highly enriched in the up-regulated gene patterns of HR patients compared with LR patients (Fig. 2B); HR patients were usually accompanied with remarkably higher expression of *HOXA/B* family genes compared with LR patients (Supplementary Fig. 4).

**Fig. 2:**
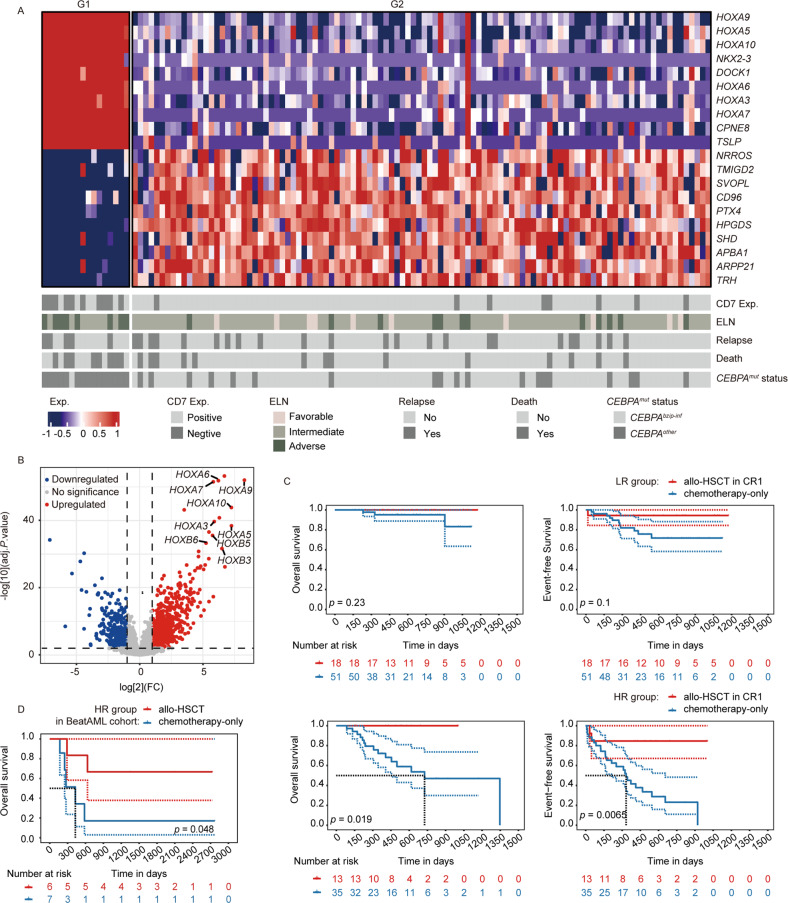
Differentially expressed genes and survival analysis of two distinct sub-cohorts of patients with CEBPAmut AML. **A** Top ten DEGs identified as up- (red) or down- (blue) regulated were ranked by the the magnitude of expression value change. **B** Volcano plot showing DEGs according to the two distinct sub-cohorts clustered by unsupervised hierarchy. **C** Kaplan–Meier curves for the survival of LR (upper panel) and HR (lower panel) *CEBPA*^*mut*^ AML patients according to treatment of allo-HSCT in CR1 or chemotherapy-only. **D** Kaplan–Meier curves for the OS of HR *CEBPA*^*mut*^ AML patients within the BeatAML cohort according to treatment of allo-HSCT or chemotherapy-only.

In addition, survival analysis revealed LR patients may not benefit from allo-HSCT in CR1, with 2-year OS and EFS of 100% and 94.4% vs. 95.2% and 71.7% in chemotherapy-only, respectively (*p* = 0.23 and 0.10, respectively); whereas allo-HSCT in CR1 could significantly improve the outcome of HR patients, with 2-year OS and EFS of 100% and 84.6% vs. 53.6% and 23.1% in chemotherapy-only, respectively (*p* = 0.019 and 0.0065, respectively) (Fig. 2C). The therapeutic efficacy of allo-HSCT was also validated in patients from BeatAML cohort. There were 13 patients eligible for the criteria of HR *CEBPA*^*mut*^ AML as we defined. The survival curves were different although the small sample size limited the statistical significance (*p* = 0.048, Fig. 2D). Therefore, not only *CEBPA*^*other*^ AML patients, *CEBPA*^*bZIP-inf*^ AML patients with negative CD7 expression or *WT1*^*mut*^ may also be recommended for allo-HSCT as soon as CR achieved.

Conclusively, CD7 immunophenotype and *WT1*^*mut*^ status is convenient for clinicians to acquire for the identification of *CEBPA*^*bZIP-inf*^ AML patients who are in risk of disease relapse (Supplementary Fig. 5). Evidences in our cohort are provided to support the necessity of allo-HSCT in CR1 for high-risk cases, with further validation in an independent cohort from BeatAML. For the limitation of the retrospective nature in this study, relative clinical trial may be conducted in the future to validate our results and explore the therapeutic efficacy of allo-HSCT in *CEBPA*^*mut*^ AML.

## Supplementary information

Supplementary Information

## Data Availability

The data that support the findings of this study are available from the corresponding author upon reasonable requests. More details are provided in Supplementary Information.
