# Supplementary material for: Characteristic immunophenotype and gene co-mutational status orchestrate to optimize the prognosis of CEBPA mutant acute myeloid leukemia
Source: Blood Cancer J. 2023 May 5;13(1):70. doi: 10.1038/s41408-023-00838-2 (PMC10162953; doi:10.1038/s41408-023-00838-2)
Supplement: Supplementary file 1 — Supplementary Information [file 41408_2023_838_MOESM1_ESM.pdf]

Characteristic immunophenotype and gene co-mutational status orchestrate to optimize the prognosis of *CEBPA* mutant acute myeloid leukemia

Supplementary Information

Supplementary Methods

Patients

In this study, we analyzed 293 consecutive *de novo* AML patients with *CEBPA* mutations. Most of the individuals (n= 228, 77.8%) were enrolled in prospective studies, involving RJ-AML 2014 Trial (n= 73, ChiCTR-OPC-15006085) (1), RJ-AML 2016 Trial (n= 120, ChiCTR-OIC-16007764) and RJ-OLD AML 2016 Trial (n= 35, ChiCTR-OIN-16008955). The remaining 65 patients were recruited to Ruijin registry and biorepository. Among all the patients, targeted NGS were performed in 124 individuals. The amino acid changes in the CEBPA protein structure were visualized using the ProteinPaint (https://pecan.stjude.org/proteinpaint). Detailed treatment procedure was depicted in Supplementary Figure 5. For our results validation, data from BeatAML cohort was downregulated from BeatAML 2.0 project's data repository (https://biodev.github.io/BeatAML2/).

This study was approved by the Ethics Committee of Ruijin Hospital Affiliated to Shanghai Jiao Tong University School of Medicine. Informed consent for both

treatment and cryopreservation of bone marrow and peripheral blood samples was obtained in accordance with the Declaration of Helsinki from all participants.

Materials and sequencing analysis

Materials investigated in this study were obtained at the time of diagnosis. The genomic DNA (isolated with DSP DNA Mini Kit, QIAGEN) and total RNA (isolated with TRIzol, Life Technologies) was extracted from mononuclear cells of patients with ≥20% blasts in bone marrow or peripheral blood according to the manufacturer's instructions.

Profiling of mutations was performed by hybrid capture-based targeted exome sequencing (TES) covering 100 genes frequently mutated in acute leukemia. TES libraries were prepared using the NadPrep EZ DNA Library Preparation Kit (Nanodigmbio), and sequencing was performed on a NextSeq 550 platform (Illumina). Paired-end reads were aligned to the hg19 reference genome. Variant calling between pairs was performed with GATK4 Mutect2, VarDict (v1.5.8), and MuTect (v1.1.7). All the mutations were annotated by snpEff (v4.2) and ANNOVAR. Homemade pipelines were used to filter SNVs and indels detected by the aforementioned software, according to the analysis standards as described previously (2). R package ComplexHeatmap (v2.15.1) was used for depicting the distribution of co-mutations.

RNA sequencing (RNA-Seq) libraries were constructed using the KAPA RNA HyperPrep kit (Roche) and sequenced on the NovaSeq 6000 platform (Illumina)

according to the manufacturer's instructions. SeqCap EZ Human Exome v3.0 kit (Roche) was used for the preparation of whole exome sequencing (WES) libraries following by sequencing on the NovaSeq 6000 platform (Illumina). The raw RNA-seq reads were mapping to human genome reference (hg19) with STAR, then featureCounts (v2.0.1) was used for the calculation of counts matrix. All gene expression data from the RNA-seq experiment were normalized using "varianceStabilizingTransformation" function from R package DESeq2 (v1.34.0), which was used as gene expression matrix for following analysis. Unsupervised clustering and CD7-supervised clustering of all 122 sample was conducted in the R package cola. Differential Gene Expression analysis was done by R package limma (v3.50.3), following the official standard workflow. The enrichment of both downregulated and upregulated genes between the groups was conducted by R package clusterProfiler (v4.2.2). The Oncoplots were drawn using R package ComplexHeatmap (v2.13.2). A total of 17385 protein-coding genes were used to analyze differential expression between the CD7-positive group and CD7-negtive group using the limma package (v.3.50.3). Gene Set Enrichment Analysis (GSEA) was performed using the "gsePathway" function in the R package ReactomePA (v.1.38.0).

Flow Cytometry

Lymphocytes in the samples were gated according to the antigen profile, scatter properties and bright positivity for CD45 (Krome Orange-conjugated, Beckman

Coulter, # B36294). Data were only accepted without lymphocyte contamination. CD7 (APC–Alexa Fluor 750-conjugated, Beckman Coulter, #B16892) was regarded as positive when at least 20% of gated cells were more fluorescent than the isotype-matched negative control. Multiparameter flow cytometry (MFC) based on a 10-color immunophenotyping panel of monoclonal antibodies against specific cell surface markers were used for distinguishing normal cells from leukemic blasts, identifying the leukemia-associated aberrant immunophenotype (LAIP) as previously described (3).

Statistical analysis

For clinical variables analysis, χ^2- or a 2-side Fisher's exact test was used for categorical variables comparison while the nonparametric Mann-Whitney U test was applied for continuous variables.

For survival analysis, overall survival (OS) defined as the time from diagnosis to death due to any cause, event-free survival (EFS) defined as the time from diagnosis to the first treatment failure, including induction failure, relapse in any site, death of any cause or development of a second tumor，and disease-free survival (DFS) defined as the time from end of induction for patients who achieved complete remission (CR) until relapse or death, was estimated using the Kaplan-Meier method and compared using the log-rank test, respectively.

For multivariate regression analysis of clinical prognostic factors, Cox-proportional hazard regression models were used. Net reclassification

improvement (NRI) calculation was performed as previously described (4) using the nricens package (version 1.6). All statistical analyses were performed with the SPSS software package, version 26 (SPSS, Chiago, IL) and R version 3.5.3 (htts://www.R-project.org/). *P*-values < 0.05 were considered significant throughout the manuscript.

Supplementary Figures and Tables

Supplementary Figure 1

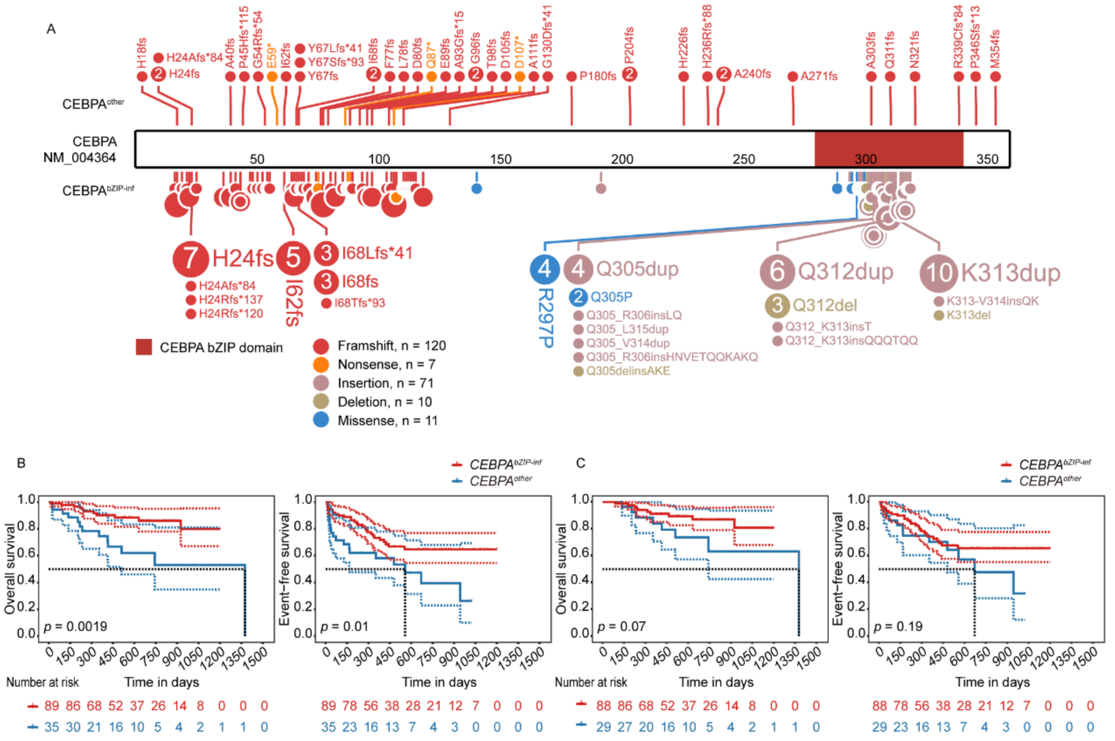

Supplementary Figure 1. The protein schematic diagram and survival curves of *CEBPA^{mut}* AML patients in our cohort.

(A) The distribution of *CEBPA* mutations whithin the cohort of 124 *CEBPA^{mut}* AML patients having available targeted sequencing data. The mutation loci of *CEBPA$^{bZIP\text{-}inf}$* and *CEBPA^{other}* was depicted below and above the protein schematic diagram, respectively.

(B) Kaplan-Meier curves for OS and EFS within the cohort of 124 *CEBPA^{mut}* AML patients.

(C) Kaplan-Meier survival curves for OS and EFS within the cohort of 117 *CEBPA^{mut}* AML patients who achieved CR during induction therapy.

Supplementary Figure 2

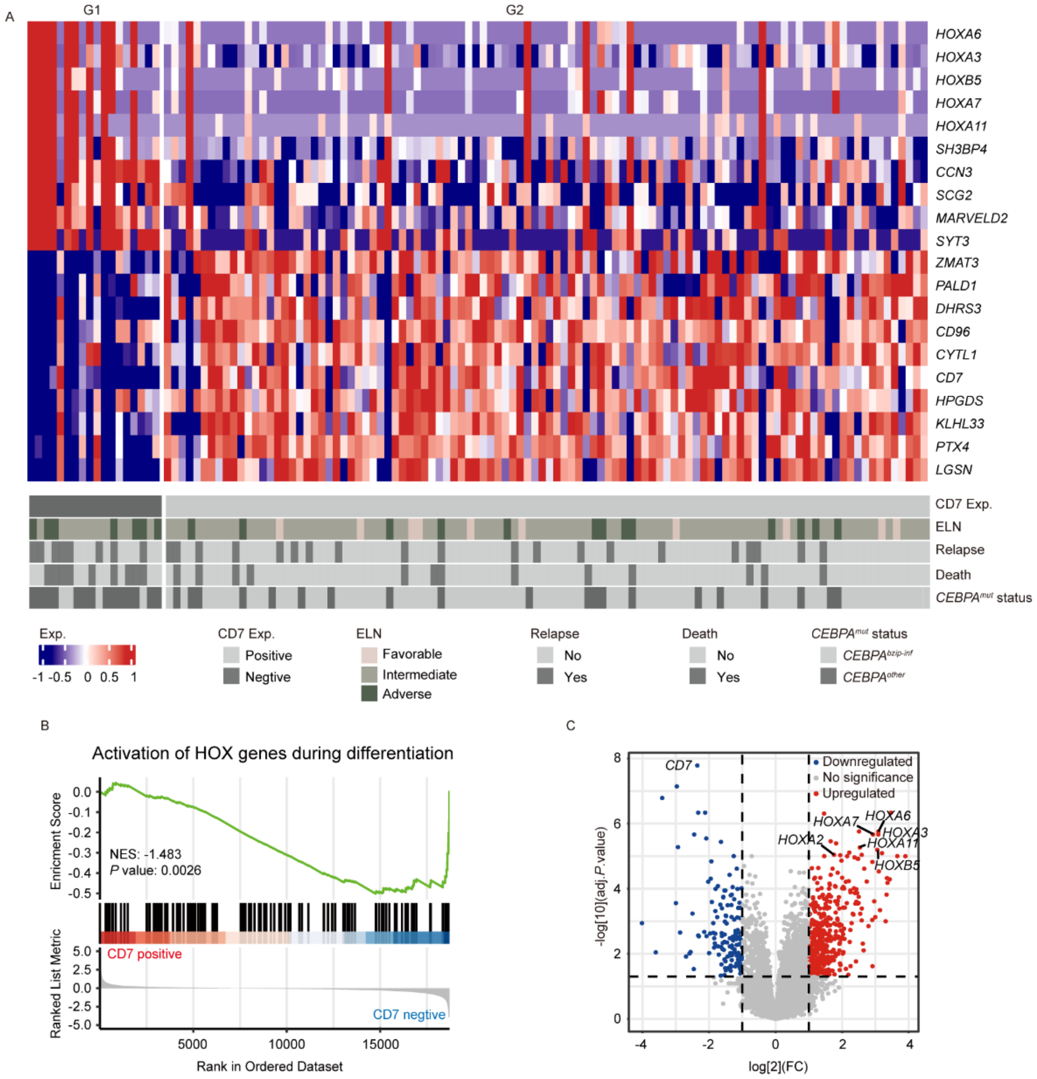

Supplementary Figure 2. CD7 positive cases showed distinct gene expression patterns compared with CD7 negative cases.

(A) Top ten DEGs identified as up- (red) or down- (blue) regulated were ranked by the the magnitude of expression value change.

(B) Gene Set Enrichment Analysis for the DEGs derived from CD7-positive cases compared to CD7-negative cases. NES, nominal enrichment score.

(C) Volcano plot showing DEGs according to the two distinct subcohorts clustered by CD7 -positive and -negative expression.

Supplementary Figure 3

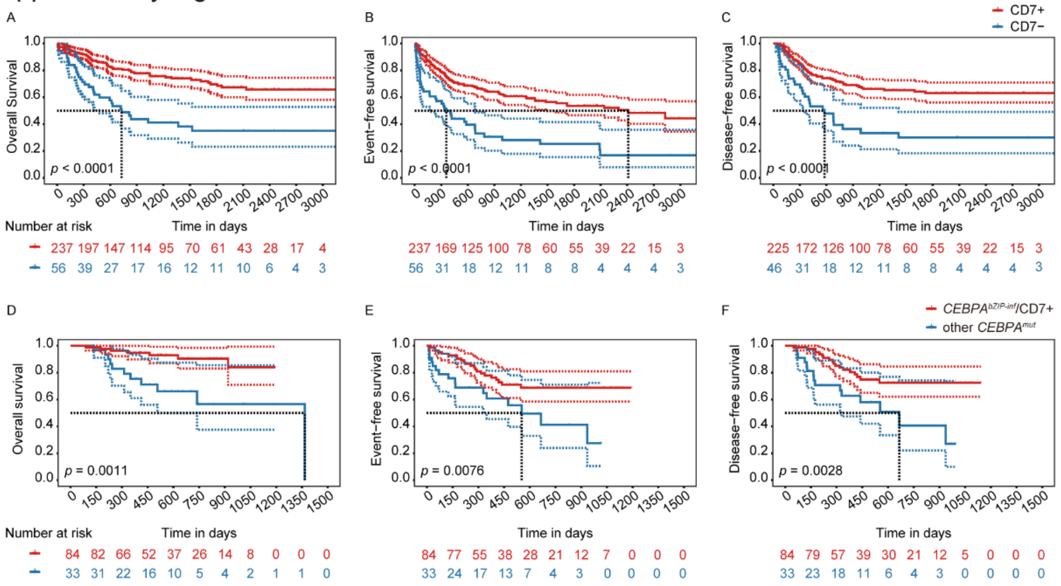

Supplementary Figure 3. Kaplan-Meier curves for the survival according to CD7 expression and *CEBPA* mutation status.

In the cohort of 293 *CEBPA*^{mut} AML patients, Kaplan-Meier curves were plotted for OS (A), EFS (B) and DFS (C) according to CD7 expression.

In the cohort of 117 CR-achieved *CEBPA*^{mut} AML patients, Kaplan-Meier curves were plotted for OS (D), EFS (E) and DFS (F) according to both CD7 expression and the mutation status of *CEBPA*.

Supplementary Figure 4

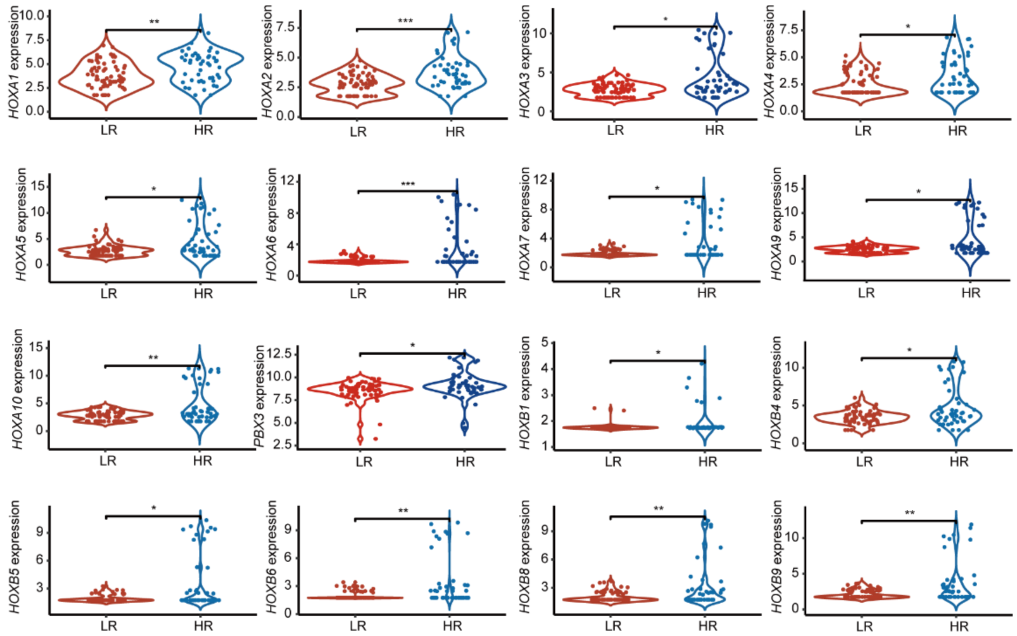

Supplementary Figure 4. The expression of *HOXA/B* family genes between the revised risk groups.

LR, the revised low-risk group; HR, the revised high-risk group.

Supplementary Figure 5

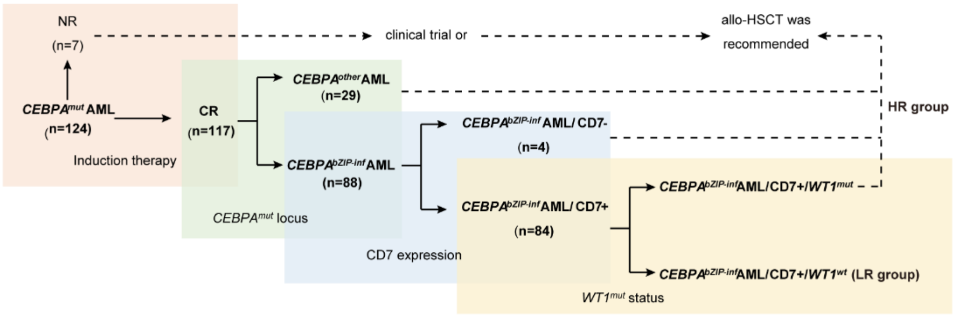

Supplementary Figure 5. Scheme of survival analysis in 124 *CEBPA^{mut}* AML patients.

Supplementary Figure 6

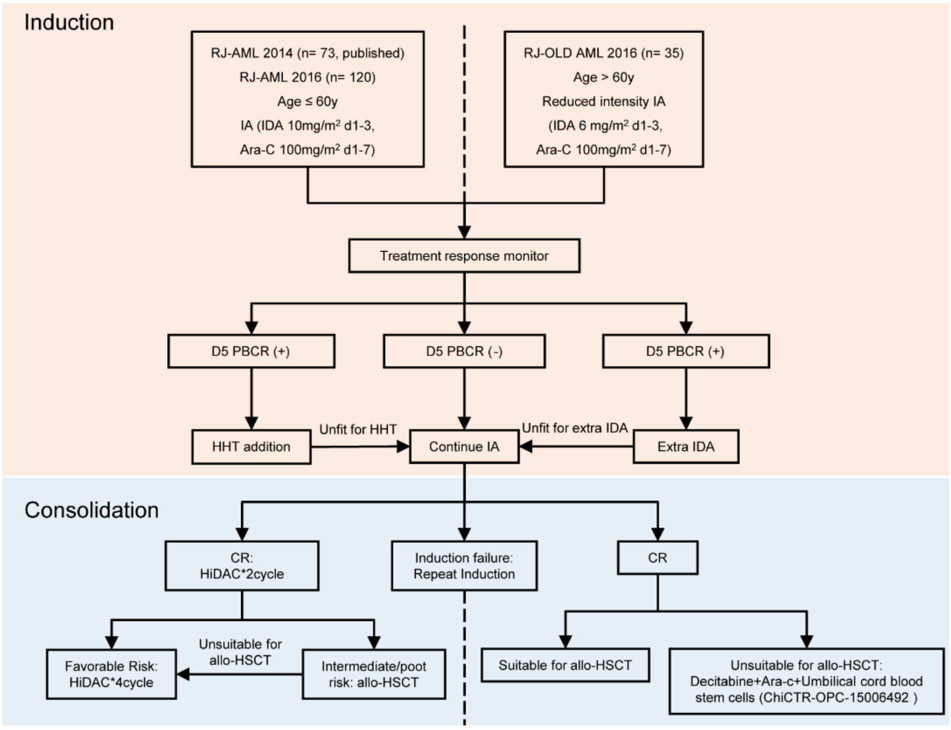

Supplementary Figure 6. Treatment protocols of the 293 *CEBPA^{mut}* AML patients enrolled in this study.

IDA, idarubicin; Ara-C, cytarabine; D5 PBCR (-), the day 5 peripheral blast clearance rate ≥ 99.55%. HHT, homoharringtonine.

Supplementary Table1: Clinical and cytogenetic characteristics of *CEBPA* $^{bZIP-inf}$ compared with *CEBPA* ^{other} AML patients.

Variable	*CEBPA* $^{bZIP-inf}$ (N=89)	*CEBPA* ^{other} (N=35)	***P*. value**
**Gender(n,%)**			
**Male**	59(66.3%)	21(60.0%)	0.537
**Female**	30(33.7%)	14(40.0%)	
**Age in years, median (IQR)**	44 (35-56)	59 (47-65)	<0.001
**WBC (*10^9/L), median (IQR)**	22 (6.68-73.4)	5.2 (2.85-21.9)	0.001
**Hb (g/L), median (IQR)**	97 (74.0-112.0)	80 (68.0-99.0)	0.041
**PLT (*10^9/L), median (IQR)**	24 (12.0-46.0)	35 (24.5-70.5)	0.01
**BM(%), median (IQR)**	61 (43.0-76.5)	55 (36.8-75.5)	0.338
**ELN, karyotype (n,%)**			
**Intermediate**	82 (92.1%)	30 (85.7%)	0.3166
**Adverse**	7 (7.9%)	5 (14.3%)	
**CD7 expression (n,%)**	85 (95.5%)	20 (57.1%)	<0.001
**CR at EOI (n,%)**	81 (91.0%)	26 (74.3%)	0.021
**CR1 (n,%)**	88 (98.9%)	29(82.9%)	0.002
**Relapse (n,%)**	21 (23.9%)	9 (31.0%)	0.818

BM, bone marrow; EOI, end of induction; CR1, first complete remission.

Supplementary Table 2. Multivariable analysis of *CEBPA* $^{bZIP-inf}$ with CD7-positive expression as a prognostic marker for OS, EFS and DFS.

Variable	Overall survival		Event-free survival		Disease-free survival	
	HR [95%CI]	*p*.val	HR [95%CI]	*p*.val	HR [95%CI]	*p*.val
CEBPA $^{bZIP-inf}$ with CD7+	0.16 [0.06, 0.49]	0.001	0.45 [0.21, 0.94]	0.034	0.39 [0.18, 0.85]	0.018
Age	1.01 [0.97, 1.04]	0.740	1.01 [0.98, 1.03]	0.505	1.01 [0.98, 1.03]	0.644
WBC	1.21 [0.37, 3.98]	0.752	1.35 [0.63, 2.89]	0.442	1.32 [0.58, 2.99]	0.510
HB	1.02 [0.99, 1.04]	0.151	1.00 [0.99, 1.01]	0.854	1.00 [0.99, 1.02]	0.790
PLT	0.99 [0.98, 1.01]	0.411	1.00 [1.00, 1.01]	0.249	1.01 [1.00, 1.01]	0.078

Supplementary Table 3. The distribution of co-mutations within 117 *CEBPA*^{mut} AML patients who achieved CR during induction therapy.

	Gene	*p*	Total (n=117)				*CEBPA*$^{bZIP\text{-}inf}$/CD7+ (n=84)				other *CEBPA*^{mut} (n=33)				*P*
			n	frequency (%)	N	Frequency (%)	n	frequency (%)	N	Frequency (%)	n	frequency (%)	N	Frequency (%)	
	GATA2	0.034	22	18.8			20	23.8			2	6.1			
**Transcriptional Factors**	***IKZF1***	1	10	8.5	31	26.5	7	8.3	27	32.1	3	9.1	4	12.1	0.027
	RUNX1	1	3	2.6			2	2.4			1	3			
	EZH2	0.442	9	7.7			8	9.5			1	3			
	ASXL1	0.067	4	3.4			1	1.2			3	9.1			
	STATG2	0.001	5	4.3			0	0			5	15.2			
	BCOR	0.006	4	3.4			0	0			4	12.1			
	SRSF2	0.078	2	1.7			0	0			2	6.1			
	ZRSR2	0.282	1	0.9			0	0			1	3			
	U2AF1	0.282	1	0.9			0	0			1	3			
	RAD21	0.097	7	6			3	3.6			4	12.1			
**Chromatin/ Cohesion/ Spliceosome**	***SMC1A***	0.135	5	4.3	54	46.2	2	2.4	34	40.5	3	9.1	20	60.6	0.049
	BCORL1	0.282	1	0.9			0	0			1	3			
	SMC3	0.558	3	2.6			3	3.6			0	0			
	TET2	0.271	18	15.4			11	13.1			7	21.2			
	DNMT3A	1	11	9.4			8	9.5			3	9.1			
	IDH2	<0.001	6	5.1			0	0			6	18.2			
	IDH1	0.316	4	3.4			2	2.4			2	6.1			
	DHX15	0.558	3	2.6			3	3.6			0	0			
	EP300	0.182	6	5.1			6	7.1			0	0			
	CSF3R	0.723	10	8.5			8	9.5			2	6.1			
	NRAS	1	15	12.8			11	13.1			4	12.1			
**Receptor Tyrosine Kinases**	***FTL3-ITD***	0.502	11	9.4	49	41.9	7	8.3	35	41.7	4	12.1	14	42.4	0.9404
	JAK3	0.107	13	11.1			12	14.3			1	3			
	KIT	1	7	6			5	6			2	6.1			
	PTPN11	0.191	3	2.6			1	1.2			2	6.1			
**Tumor supressors**	***WT1***	-	-	-	23	19.7	-	-	15	17.9	-	-	8	24.2	0.4342
**Nucleolar**	***NPM1***	-	-	-	7	6	-	-	0	0	-	-	7	21.2	<0.001

p value indicated the difference significance of indicated genes between *CEBPA*$^{bZIP\text{-}inf}$/CD7+ and other *CEBPA*^{mut} AML cases；

P value indicated the difference significance of indicated gene groups between *CEBPA*$^{bZIP\text{-}inf}$/CD7+ and other *CEBPA*^{mut} AML cases.

References:

1. Zhang Y, Li X, Weng X, Shen Y, Chen Y, Zheng Y, et al. Optimization of idarubicin and cytarabine induction regimen with homoharringtonine for newly diagnosed acute myeloid leukemia patients based on the peripheral blast clearance rate: A single-arm, phase 2 trial (RJ-AML 2014). American journal of hematology. 2021;97(1):43-51.
2. Cheng WY, Li JF, Zhu YM, Lin XJ, Wen LJ, Zhang F, et al. Transcriptome-based molecular subtypes and differentiation hierarchies improve the classification framework of acute myeloid leukemia. Proceedings of the National Academy of Sciences of the United States of America. 2022;119(49):e2211429119.
3. Liu FJ, Cheng WY, Lin XJ, Wang SY, Jiang TY, Ma TT, et al. Measurable Residual Disease Detected by Multiparameter Flow Cytometry and Sequencing Improves Prediction of Relapse and Survival in Acute Myeloid Leukemia. Front Oncol. 2021;11:677833.
4. Alba AC, Agoritsas T, Walsh M, Hanna S, Iorio A, Devereaux PJ, et al. Discrimination and Calibration of Clinical Prediction Models: Users' Guides to the Medical Literature. Jama. 2017;318(14):1377-84.
